# Prevalence of Hepatitis B Infection Among Pregnant Women in Oman

**DOI:** 10.1007/s44197-022-00043-7

**Published:** 2022-05-28

**Authors:** Omaima Mohamed Al-Ismaili, Amina Al-Jardani, Fatma Al-Hinai, Intisar Al-Shukri, Mersum Mathew, Seif Al-Abri, Hanan Al-Kindi

**Affiliations:** 1 Royal Hospital, Ministry of Health, Muscat, Oman; 2 Central Public Health Laboratory, Directorate General for Disease Surveillance and Contro, Ministry of Health, Muscat, Oman; 3 Directorate General for Disease Surveillance and Control, Ministry of Health, Muscat, Oman; 4 Directorate General of Primary Health Care, Ministry of Health, Muscat, Oman

**Keywords:** Heptitis B virus, Oman, Pregnant, Prevalence, Transmission

## Abstract

**Objective:**

The primary objective of our study was to estimate the prevalence of hepatitis B infection among pregnant women in Oman. The secondary objectives were to test for other hepatitis B virus (HBV) serological markers and to determine hepatitis B viral load.

**Methods:**

In this cross-sectional study conducted from June 2019 to December 2020, we randomly selected 2210 Omani women who attended antenatal clinics across the country. Pregnant women were tested for HBV surface antigen (HBsAg) using a commercial enzyme immunoassay; those who tested positive were further analyzed for other HBV serological markers: HBV core total antibody (anti-HBc), HBV core antibody IgM (immunoglobulin M) (anti-HBc IgM), hepatitis B virus e antigen (HBeAg) and hepatitis B virus e antibody (anti-HBe). They were also tested for hepatitis B viral load.

**Results:**

This study included 2210 women with a mean age of 39 years. Ninety-five percent of the women who were invited to participate consented and were included in the study. Thirty-three (1.49%) tested positive for HBsAg. All participants tested negative for HBeAg and anti-HBc IgM and positive for anti-HBc and anti-HBe, and 30 (90.9%) women had their hepatitis B viral load measured. Three (10.0%) had undetectable viral loads, 23 (76.7%) had low viral loads of < 2000 (IU/ml), 2 had moderate viral loads between 2000 and 200,000 (IU/ml) and one had a high viral load of 486,000 (IU/ml).

**Conclusion:**

Our study shows that the HBV prevalence in pregnant women is 1.49%, which is lower than what was reported earlier. Nevertheless, antenatal screening is still warranted, as there are vital interventions for the newborn and the mother.

## Introduction

HBV is a major health concern affecting the lives of more than 250 million people worldwide and causing over 780,000 deaths annually. The prevalence is estimated to be highest in African (6.1%) and Western Pacific (6.2%) regions. In the Mediterranean region, it is estimated to be approximately 3.3% [1]. The prevalence of chronic HBV infection in Oman is estimated to be between 2 and 7% [2, 3].

Infection with HBV causes a wide spectrum of clinical presentations. It can cause asymptomatic infection, acute hepatitis, chronic hepatitis and hepatocellular carcinoma [4]. Chronic hepatitis B has different manifestations. Approximately 30% of people with chronic hepatitis B will have serious consequences [5]; this includes liver cirrhosis, end-stage liver failure and hepatocellular carcinoma. The virus genotype and the viral DNA in blood, as well as some patient factors (age, sex, genetic factors, immune status), seem to determine who will develop these outcomes. The prognosis of chronic hepatitis B infection depends on the severity of liver injury [6].

Perinatal transmission is one of the most efficient modes of HBV transmission. It often leads to severe long-term sequelae. The percentage of infected (HBV e antigen (HBeAg)-positive) infants who are born to a mother who is positive for HBV surface antigen (HBsAg) is approximately 70–90%. On the other hand, the percentage of infected infants who are HBeAg-negative born to mothers who are HBsAg-positive is approximately 5–20% [7]. Of the infants infected, approximately 90% will develop chronic hepatitis B, of whom 25% are at risk of death due to liver failure or hepatocellular carcinoma [8–10].

Studies regarding the prevalence of hepatitis B in the Omani population are limited, with no recent studies reflecting the current situation [2, 9, 10]. A study on seropositivity by Al-Dhahry et al. in 1994 showed that hemodialysis patients had an HBsAg prevalence of 12.7% compared to 4.5% in healthy subjects [9]. This was a single-center study on patients at higher risk of contracting hepatitis B infection than healthy individuals. In 2006, Awaidy et al. estimated HBV sero-prevalence in pregnant women in Oman, Qatar and the UAE. Their results showed that 7% of the women in Oman were HBsAg-positive [10]. Both studies place Oman among countries with intermediate endemicity of hepatitis B. As a result, many measures, including hepatitis B vaccination and screening of high-risk patients, were introduced, with no subsequent studies to audit the impact of these measures.

Knowing the prevalence, especially among pregnant women, encourages the introduction of antenatal screening, which aids in diagnosing mothers and planning interventions for mothers and newborns. It includes starting treatment in pregnant mothers during the second or third trimester if the viral load is high (> 200,000 IU/ml) [11]. For newborns, interventions include hepatitis B immunoglobulin intramuscular injection, timely immunization completion and proper follow-up [12]. These interventions prevent the development of persistent HBV infection in over 90% of these cases [11]. The primary aim of this study was to determine the prevalence of hepatitis B infection among pregnant women in Oman. The secondary objectives were to test for other HBV serological markers and to determine hepatitis B viral load.

## Methodology

We conducted a cross-sectional study of pregnant Omani women from June 2019 to December 2020.

### Population

The target population was pregnant Omani women presenting to local health centers across all governorates for registration, irrespective of gestational age. Pregnant women who did not provide consent were excluded from the study.

### Sample Size and Sampling Method

The sample size was calculated based on the estimated prevalence of hepatitis B infection in Oman of approximately 3% [3, 10], a population size of 89,336 [13], an absolute precision of 1%, a confidence level of 95% and a design effect of 2 (multistage cluster random sampling).

The sample size was 2209 (calculated with an online calculator, OpenEpi).

To represent pregnant women throughout Oman, we used multistage cluster random sampling. In the first stage, a proportional number of women from each governorate were selected based on the number of women attending antenatal clinics in that specific governorate.

In the second stage, one health center from each governorate was randomly selected so that women from all governorates would be included. Then, in that health center, alternative consecutive women were selected.

### Process

Blood samples were drawn during the antenatal visits by the attending nurse in that clinic after the patient consented to participate. Then, the blood sample was sent to the Central Public Health Laboratory, where it was tested. Serum samples were separated and stored at − 20 °C. Testing for HBsAg was performed using an enzyme immunoassay (Monolisa™ HBsAg ULTRA, Bio-Rad, France). The platform was validated internally using positive and negative controls with each run. In addition, in-house positive controls were used.

Samples that tested positive for HBsAg were first confirmed with a neutralization assay (Monolisa™ HBs Ag ULTRA Confirmatory, Bio-Rad, France) and then tested for other serological markers (HBeAg, anti-HBe and anti-HBc IgM). If HBsAg was detected, the patient was called for counseling and liver function tests, including alanine aminotransferase (ALT) tests, and another sample for viral load tests was taken. Samples were sent to the Central Public Health Laboratory and tested for HBV viral load by real-time polymerase chain reaction (Cobas 4800, Roche, Basel, Switzerland).

### Data Analysis

Prevalence is presented as a percentage with a 95% confidence interval (CI). All analyses were performed using the Statistical Program for Social Sciences (SPSS, IBM, Armonk, NY).

This study was approved by the Research and Ethical Review Board of the Ministry of Health, Oman, and signed informed consent was obtained from all the participants.

## Results

This study included 2210 women. The median age of the included subjects was 30 years, with a range from 16 to 49 years. Four hundred and thirty women (19.5%) were primigravida (first pregnancy). The median number of previous children was 2, with a maximum of 12 children.

Of the 2210 enrolled women, 33 tested positive for HBsAg (prevalence 1.49%, 95% CI 1.1–2.1%). All women tested negative for HBeAg and anti-HBc IgM and positive for anti-HBc and anti-HBe. The distribution of women included in the study (by governorate) and the prevalence of HBsAg are presented in Table 1.

**Table 1:** Distribution of HBsAg-seropositive women by governorate

Governorate	Number of women included	Number of women seropositive for HBsAg	Percentage of women seropositive for HBsAg
Muscat	466	4	0.85
Dhofar	199	3	1.51
Musandam	20	0	0
Al Buraymi	51	0	0
Ad Dakhiliyah	296	6	2.03
North Al Batinah	424	6	1.42
South Al Batinah	261	3	1.15
North Ash Sharqiyah	155	5	3.23
South Ash Sharqiyah	188	4	2.13
Ad Dhahirah	126	2	1.59
Al Wusta	24	0	0
**Total**	**2210**	**33**	**1.49**

### Hbsag-Positive Women

The median age of HBsAg-seropositive women was 34 years (24–43 years). The majority, 84.8%, of HBsAg-seropositive women were 30 years or older. The mean number of children was 2 (1–11 children), and 64.6% of HBsAg-seropositive women had 3 or more children. Most participants, 28/33 (84.8%), had their ALT levels measured, and 30 women had their HBV viral loads evaluated. Missing results were due to participants being lost to follow-up. Only two women (7.1%) had elevated ALT levels (Table 2). Only two women (6.7%) had moderate viral loads, and one had a high viral load (Table 2). Women who tested positive for HBsAg were referred to a gastroenterologist in their governorate for counseling, follow-up and further management planning for them and their newborns.

**Table 2:** ALT level and HBV viral load results of HBsAg-seropositive women

Number of women tested for ALT	28	Number of women tested for hepatitis B viral load	30
Number of women who had normal ALT levels (7–40 IU/L)	26	Number of women who had a detectable viral load	27
Number of women who had ALT × 1 upper normal limit	2	Number of women who had a viral load between:	
		0 and 1999 (IU/ml)	23
		2000 and 200,000 (IU/ml)	3
		> 200,000 (IU/ml)	1

Only 4/33 (12.1%) women were known to have HBV infections. They are being followed up in tertiary hospitals in the Muscat governorate. They had a low viral load and were not on antiviral therapy.

## Discussion

This cross-sectional study was conducted on 2210 pregnant Omani women to estimate the prevalence of HBV. The prevalence among pregnant women was 1.49%. This prevalence is similar to what is reported in neighboring countries. Alrowaily and colleagues reported a prevalence of 1.6% in Saudi Arabia [14]. A prevalence of 1.5% in the UAE and 1% in Qatar was reported in a cross-sectional study in 2006 [10]. In Bahrain, the prevalence was estimated to be 0.58% [15]. Other countries in the Mediterranean region show similar results as well. The prevalence is estimated to be 1.2% in Iran and 2.1% in Turkey [16]. Limitations of the abovementioned studies were the sampling techniques and the sample sizes.

Our result is much lower than the 7.1% prevalence described in 2006. Awaidy et al. studied 604 pregnant women from 6 centers in Oman. Forty-three women (7.1%) tested positive for HBsAg, and 3 of these women were also HBeAg-positive [10]. This finding indicates that Oman has a low HBV prevalence (< 2%) as opposed to the intermediate prevalence that was reported in earlier studies [9, 10]. This finding might be explained by the introduction of the HBV vaccine as part of the Expanded Program of Immunization in the country in 1990, and the generation of women who received the HBV vaccine is now of childbearing age. Additionally, this is likely the reason why most infected women in the present study were 30 years or older. The results of this study reflect the precautionary measures that were introduced, including the introduction of hepatitis B vaccination in the Expanded Program of Immunization schedule from August 1, 1990, with the achievement of over 97% vaccine coverage. The catch-up school campaign started in 2001 and continued until 2004–2014 in Oman, and all people younger than 28 years received the hepatitis B vaccine. Immunization of spouses and family contacts of all hepatitis B patients and carriers began in 2002. The same policy of contact immunization was also followed for the contacts of HBsAg blood donors [17].

All HBsAg-seropositive women in our study were in the inactive HBsAg carrier stage. All patients were negative for HBeAg. This is different from what was found in 2006 [10], where 0.5% of women tested positive for HBeAg. All had a low viral load except for one participant who had a viral load of 486,000 IU/ml. This participant was only diagnosed through this study and would have been a candidate for antiviral therapy during pregnancy and hepatitis B immunoglobulin intramuscular injection for newborns. The course of the inactive HBsAg carrier stage is usually benign. However, this stage carries a 20–30% risk of spontaneous reactivation and liver cancer [6].

Ad Dakhiliyah and North Al Batinah had the highest number of positive cases. A larger sample size might be needed to reflect the true prevalence in each governorate. Most women were not diagnosed with hepatitis B infection before the study.

This study has some limitations. First, we calculated the sample size based on the prevalence reported from old data and in a specific population. A larger sample size might have been needed if the prevalence was lower. In addition, we did not obtain more information about the risk factors for HBV infection.

The strengths of our study include the following: first, it was a prospective study, and subjects were randomly selected from all governorates across Oman. Second, it is one of the largest studies in the Gulf area to study the prevalence of HBV in pregnant women. Third, we managed to measure all the parameters for HBV infection, such as other HBV profiles and HBV viral load.

## Conclusion

Our study shows that HBV prevalence in pregnant women in Oman is 1.49%, which is lower than what was reported earlier. Nevertheless, antenatal screening is still warranted, as there are important interventions for both newborns and mothers. This study should guide public health authorities in Oman to develop guidelines for the antenatal screening and management of HBV infection in mothers and newborns, and it will also help Oman to develop a national strategic plan for the elimination of HBV and hepatitis C virus infections.

**Advances in Knowledge**

Hepatitis B virus affects the lives of millions of patients worldwide and causes significant morbidity and mortality. Data regarding the prevalence of hepatitis B among pregnant women in Oman are limited and outdated. These findings will allow us to estimate the prevalence of hepatitis B infection among pregnant women and help to map the level of endemicity of hepatitis B in our country. This study also aimed to test for other identifiers of HBV and determine viral load.

**Application to Patient Care**

Knowing the prevalence of hepatitis B, especially in pregnant women, would help in the planning of interventions, such as initiating antenatal screening for hepatitis B infection, treating mothers with antiviral agents and providing hepatitis B immune globulin (HBIG) treatment for babies born to affected mothers, to decrease the risk of transmission.

## Data Availability

The datasets during the current study are available from the corresponding author on reasonable request.

## Abbreviations

HBV: Hepatitis B virus
HBsAg: HBV surface antigen
Anti-HBc: HBV core total antibody
Anti-HBc IgM: HBV core antibody immunoglobulin M
HBeAg: Hepatitis B virus e antigen
Anti-HBe: Hepatitis B virus e antibody
ALT: Alanine aminotransferase
CI: Confidence interval
SPSS: Statistical Program for Social Sciences
